# Supplementary figures and images for: Tumor Necrosis Factor and Schistosoma mansoni egg antigen omega-1 shape distinct aspects of the early egg-induced granulomatous response
Source: PLoS Negl Trop Dis. 2021 Jan 19;15(1):e0008814. doi: 10.1371/journal.pntd.0008814 (PMC7845976; doi:10.1371/journal.pntd.0008814)

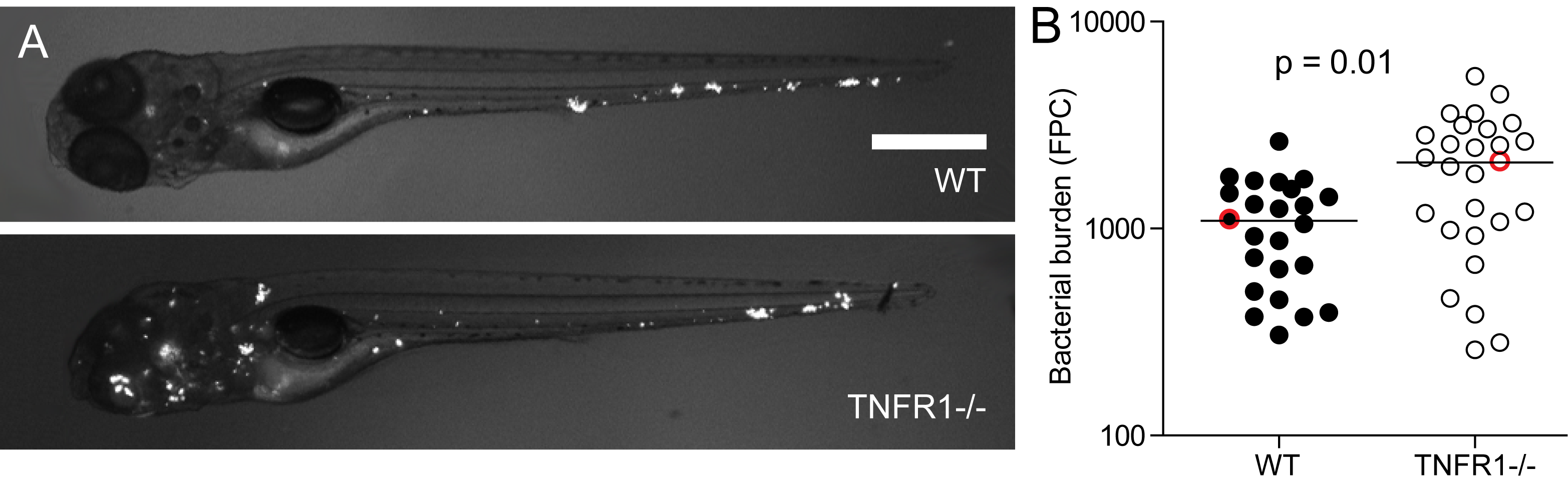

Supplement: S1 Fig — WT and TNFR1 mutant zebrafish larvae were systemically infected at 36 hours post-fertilization via caudal vein injection with 75 CFU Mycobacterium marinum, and then imaged at 4 days post-infection for bacterial burden. (A) The two animals closest to the mean. Scale bar, 300 μm. (B) Quantification of bacterial burden, with the two red data points corresponding to the animals in (A). Horizontal bar, means. Statistics, Student’s t test. FPC: fluorescent pixel counts. (TIF) [file pntd.0008814.s001.tif]
