# Supplementary material for: Tumor Necrosis Factor and Schistosoma mansoni egg antigen omega-1 shape distinct aspects of the early egg-induced granulomatous response
Source: PLoS Negl Trop Dis. 2021 Jan 19;15(1):e0008814. doi: 10.1371/journal.pntd.0008814 (PMC7845976; doi:10.1371/journal.pntd.0008814)
Supplement: Attachment — Submitted filename: Reviewer Responses.docx [file pntd.0008814.s002.docx]

**PLoS NTD response letter**

*Reviewer #1: This is a short but interesting paper, presenting the use of a novel animal platform to explore a protein released by schistosome eggs driving the formation of granuloma and more specifically macrophage recruitment. There is a large amount of background in the Results section which needs to be moved to the introduction or discussion.*

We had tried to put the necessary background information in the introduction, but it did not work. The introductory parts included in the results are specific to that set of results, so we would prefer to leave them where they are.

*The reference to an accepted paper (Takaki et al., 2020)(Cell Host & Microbe, accepted) is problematic and impossible to review, while some efforts have been to demonstrated key points (fig s1).*

Apologies, we had attached the paper in press as part of our submission in the hope that it would be passed on to you. It has just been published today: <https://www.cell.com/cell-host-microbe/fulltext/S1931-3128(20)30560-6>

We will update the manuscript to reflect this.

*In Figure 2D in omega-1 samples the two outlier significantly increase the average of this condition, what do the authors think of these samples, since this is not seen in 2B?*

We are not sure why these two points appeared in this one assay only. We have tested the data set using the Grubbs' test for outliers, and found that only the largest value, 38, was recognized as an outlier. Removing this identified outlier did not change significance. Removing both the outlier and the second highest value, 37, still retained a significant difference between the two groups (p<0.05). We are unsure what these mean, but did not think to take them out.

|  | PBS | DEPC-treated Omega-1 |
| --- | --- | --- |
| No outliers removed | *** | ** |
| 38 (outlier) removed | *** | ** |
| 38 (outlier) and 37 removed | *** | * |

*The importance of RNase activity in mac recruitment is clearly shown in DEPC treatment. 
The authors mention that a omega1 KO shipments wasn’t viable so they performed some other experiments, and then they had another shipment which is shown in 2e? This is not clear.*

We tried to obtain two shipments of Omega-1 KO eggs so we could assess granuloma formation in detail, but both shipments contained only dead eggs. This is why we used antigens derived from eggs, and recombinant omega-1. In figure 2E we used two versions of recombinant omega-1, WT and RNase mutant recombinant omega-1. Our wording in the figure legend was confusing, which we have now clarified.

*The authors comment about RNase activity with their samples, but what about cytotoxity against zebra fish cells specifically? This is important since O-1 is a major hepatotoxin. Does DEPC treat eliminated cytotoxicity also?*

We were not able to evaluate cytotoxicity. We had used Sytox staining to assess membrane permeabilization as a marker for cell damage in response to direct injection of egg antigens into the hindbrain ventricle, as well as in response to implanted eggs. However, these results were not clear, due to background staining of the hindbrain ventricle even in the absence of eggs or antigens.

At early timepoints, implantation of whole eggs appeared to induce some early periovular Sytox staining of nearby epithelial cells of the hindbrain ventricle, however, there was no obvious staining in response to direct injection of egg antigens. At later timepoints, the macrophages of the epithelioid granuloma were clearly Sytox-negative. We were unable to access nearby epithelial cells as they were all Sytox positive, even in the absence of the egg or its antigens.

Currently, we think that while omega-1 is cytotoxic to hepatocytes, that in the context of a functioning immune response, it may induce cytotoxicity below the threshold of increasing cell permeability in epithelial cells and macrophages. Based on the finding that injection of omega-1 into mice induces IL-33 expression (Hams et al., 2016), we imagine a scenario in which RNA degradation leads to an inhibition of protein synthesis which in turn perturbs cellular homeostasis, then inducing cell stress signaling, including IL-33 (DAMP/alarmin), which in turn induces macrophage recruitment and granuloma formation. These ideas can be pursued in future work as we are not in a position to do so now.

*Reviewer #2: This study uses an important and novel new model to confirm some previous findings in mice. It contains some interesting data, however their novelty is limited by the fact that both findings (the role of TNF and Omega-1 in granuloma formation) have previously been tested in mice. Furthermore, the two parts of the manuscript did not lead naturally on from one another and it appeared to be two separate studies.*

The studies were a continuum of experiments all designed to get at the host and pathogen factors mediating granuloma formation.  *Major comment:
Further work should be carried out to determine the mechanistic basis of RNAse-dependent macrophage recruitment to Omega-1. The authors propose a mechanism through epithelial cells - data should be provided to either back up this claim, or alternatively, Omega-1 could be acting directly on macrophages - the authors could address this by using WT or RNAse-deficient Omega-1 and assess whether either proteins induce isolated (human, mouse or fish) macrophage chemotaxis in vitro.*

These are excellent suggestions for future work and we very much hope that the entire community of Schistosome researchers will be enthusiastic about this facile model. As we have done for our TB colleagues, we will be very happy to induct people into the techniques and share reagents.

*Minor comment:
Page 10: "The omega-1 deficient SEA retains ~20% of the omega-1 RNase activity (not shown), suggesting that even though reduced compared to wild type eggs, it is may still be sufficient for macrophage recruitment (Ittiprasert et al., 2019)." This statement should be clarified - does the knockdown only reduce Omega-1 expression by 80% or is there RNAse activity from some other source?*

Knockout of pools of eggs with the CRISPR/Cas9 construct is not 100% efficient, with some eggs retaining their WT omega-1 sequence. Consequently, pools of eggs transduced with the CRISPR/Cas9 construct targeting omega-1 still retain approximately 20% of their WT omega-1 transcript, and likewise, SEA from omega-1 knockout eggs also retains about 20% of its RNase activity. We have now clarified this in the main text.

Hams, E., Bermingham, R., Wurlod, F.A., Hogan, A.E., O'Shea, D., Preston, R.J., Rodewald, H.R., McKenzie, A.N., and Fallon, P.G. (2016). The helminth T2 RNase omega1 promotes metabolic homeostasis in an IL-33- and group 2 innate lymphoid cell-dependent mechanism. Faseb J *30*, 824-835.
